# Preferences of inflammatory arthritis patients for biological disease-modifying antirheumatic drugs in the first 100 days of the COVID-19 pandemic

**DOI:** 10.3906/sag-2012-5

**Published:** 2021-08-30

**Authors:** Umut KALYONCU, Yavuz PEHLİVAN, Servet AKAR, Timuçin KAŞİFOĞLU, Gezmiş KİMYON, Ömer KARADAĞ, Ediz DALKILIÇ, Ali İhsan ERTENLİ, Levent KILIÇ, Duygu ERSÖZLÜ, Cemal BES, Hakan EMMUNGİL, Rıdvan MERCAN, Elif Durak EDİBOĞLU, Nilüfer KANITEZ, Emre BİLGİN, Seda ÇOLAK, Süleyman Serdar KOCA, Emel GÖNÜLLÜ, Orhan KÜÇÜKŞAHİN, Nihan COŞKUN, Burcu YAĞIZ, Sedat KİRAZ

**Affiliations:** 1 Division of Rheumatology, Department of Internal Medicine, Hacettepe University, Faculty of Medicine, Ankara Turkey; 2 Division of Rheumatology, Department of Internal Medicine, Uludağ University, Faculty of Medicine, Bursa Turkey; 3 Division of Rheumatology, Department of Internal Medicine, İzmir Katip Çelebi University, Faculty of Medicine, İzmir Turkey; 4 Division of Rheumatology, Department of Internal Medicine, Eskişehir Osmangazi University, Faculty of Medicine, Eskişehir Turkey; 5 Division of Rheumatology, Department of Internal Medicine, Faculty of Medicine, Hatay Mustafa Kemal University, Hatay Turkey; 6 Division of Rheumatology, Department of Internal Medicine, Ministry of Health Adana City Training and Research Hospital, Adana Turkey; 7 Division of Rheumatology, Department of Internal Medicine, Başakşehir Çam and Sakura City Hospital, İstanbul Turkey; 8 Division of Rheumatology, Department of Internal Medicine, Faculty of Medicine, Trakya University, Edirne Turkey; 9 Division of Rheumatology, Department of Internal Medicine, Faculty of Medicine, Tekirdağ Namık Kemal University, Tekirdağ Turkey; 10 Division of Rheumatology, Department of Internal Medicine, Faculty of Medicine, Koç University, İstanbul Turkey; 11 Division of Rheumatology, Department of Internal Medicine, Gulhane Faculty of Medicine, University of Health Sciences, Ankara Turkey; 12 Division of Rheumatology, Department of Internal Medicine, Faculty of Medicine, Fırat University, Elazığ Turkey; 13 Division of Rheumatology, Department of Internal Medicine, Faculty of Medicine, Sakarya University, Sakarya Turkey; 14 Division of Rheumatology, Department of Internal Medicine, Faculty of Medicine, Yıldırım Beyazıt University, Ankara Turkey

**Keywords:** COVID-19, biologic DMARDs, rheumatoid arthritis, spondyloarthritis

## Abstract

**Background/aim:**

To evaluate treatment adherence and predictors of drug discontinuation among patients with inflammatory arthritis receiving bDMARDs within the first 100 days after the announcement of the COVID-19 pandemic.

**Materials and methods:**

A total of 1871 patients recorded in TReasure registry for whom advanced therapy was prescribed for rheumatoid arthritis (RA) or spondyloarthritis (SpA) within the 3 months (6–9 months for rituximab) before the declaration of COVID-19 pandemic were evaluated, and 1394 (74.5%) responded to the phone survey. Patients’ data regarding demographic, clinical characteristics and disease activity before the pandemic were recorded. The patients were inquired about the diagnosis of COVID-19, the rate of continuation on bDMARDs, the reasons for treatment discontinuation, if any, and the current general disease activity (visual analog scale, [VAS]).

**Results:**

A total of 1394 patients (493 RA [47.3% on anti-TNF] patients and 901 SpA [90.0% on anti-TNF] patients) were included in the study. Overall, 2.8% of the patients had symptoms suggesting COVID-19, and 2 (0.15%) patients had PCR-confirmed COVID-19. Overall, 18.1% of all patients (13.8% of the RA and 20.5% of the SpA; p = 0.003) discontinued their bDMARDs. In the SpA group, the patients who discontinued bDMARDs were younger (40 [21–73] vs. 44 years [20–79]; p = 0.005) and had higher general disease activity; however, no difference was relevant for RA patients.

**Conclusion:**

Although the COVID-19 was quite uncommon in the first 100 days of the pandemic, nearly one-fifth of the patients discontinued bDMARDs within this period. The long-term effects of the pandemic should be monitored.

## 1. Introduction

The recent coronavirus disease 2019 (COVID-19) pandemic is caused by severe acute respiratory syndrome coronavirus 2 (SARS-CoV-2). Fever, dry cough, sore throat, and muscle and joint pain are general disease manifestations, and a severe clinical picture requiring hospital admission is encountered in 15%–20% of the patients [1,2]. According to the data collected from various countries, the COVID-19 fatality rate is about 1%–10% [3]. Patients with inflammatory arthritis such as rheumatoid arthritis (RA) and spondyloarthritis (SpA) regularly and continuously receive synthetic or biological disease-modifying antirheumatic drugs (DMARDs) as the main component of their treatment. On the other hand, temporary discontinuation of particularly biological DMARDs (bDMARDs) in the presence of infection is an accepted recommendation [4]. Currently, the world is reexperiencing a pandemic after a period of nearly 100 years. After March 11, 2020, when the World Health Organization announced the pandemic, American, European, and local societies of rheumatology have claimed general recommendations about drug usage [4–6]. The Turkish Society for Rheumatology released COVID-19 recommendations on March 27, 2020, and left the decision to use synthetic/biological DMARDs during the pandemic mainly to the primary physician that follows the patient [6]. On the other hand, the behavioral pattern of patients with inflammatory arthritis using biological/synthetic DMARDs during the COVID-19 pandemic is unknown in Turkey or the rest of the world. 

Accordingly, the present study aimed to evaluate treatment adherence of patients with inflammatory arthritis receiving bDMARDs within the first 100 days after the announcement of the COVID-19 pandemic.

## 2. Methods

### 2.1. Patient selection

The TReasure registry is a web-based, prospective, observational cohort including RA and spondyloarthritis (SpA) patients from 17 centers in different regions of Turkey and was established in December 2017. Details of the establishment of TReasure registry were previously reported [7]. As of March 2020, there were a total of 7471 patients with inflammatory arthritis (2560 RA patients and 4911 SpA patients) receiving bDMARDs in this registry. The bDMARDs were as follows (arranged alphabetically): abatacept, adalimumab, certolizumab, etanercept, golimumab, infliximab, rituximab, secukinumab, and tocilizumab. 

The present study included patients who were prescribed bDMARDs and for whom disease activity was recorded within the 3-month period before the declaration date of pandemic (March 2020) in the TReasure registry. For rituximab therapy, this period was determined to be 6–9 months before the declaration date of the pandemic. In the TReasure registry, the target population consisted of 1871 patients, of whom 1394 (74.5%) completed the standard phone questionnaire, 39 (2.1%) refused to participate in the study, and the remaining could not be reached. The patients who participated and those who did not participate in the study did not differ in demographic and clinical characteristics (data not shown).

### 2.2. Demographic and clinical characteristics of the patients

The demographic and clinical data collected from the patients were defined previously [7]. In brief, the following data were recorded for both RA and SpA patients: age, sex, disease duration, comorbidities (the Charlson comorbidity index), erythrocyte sedimentation rate (ESR) (mm/h), C-reactive protein (CRP) level (mg/L), number of swollen (66 joints) and tender (68 joints) joints, visual analog scale (VAS)-pain score, patients’ global assessment-VAS, and VAS-fatigue score, and the names of the currently used synthetic DMARDs or bDMARDs. Additionally, in RA patients, positivity for rheumatoid factor (RF) and anticyclic citrullinated peptide (anti-CCP) was determined, and the scores of the disease activity score-28 (DAS-28), the Crohn’s Disease Activity Index (CDAI), the Simple Disease Activity Index (SDAI), and the Health Assessment Questionnaire (HAQ) were calculated to assess disease activity. In SpA patients, the Bath Ankylosing Spondylitis Disease Activity Index (BASDAI), the Bath Ankylosing Spondylitis Functional Index (BASFI), and the Ankylosing Spondylitis Disease Activity Score (ASDAS) based on CRP (ASDAS-CRP) were used for the assessment of disease activity. The diagnoses in SpA patients were classified as ankylosing spondylitis (according to the modified New York criteria), nonradiographic SpA (according to the axial SpA criteria), peripheral SpA (according to the peripheral SpA criteria), psoriatic arthritis (according to the CASPAR criteria), and enteropathic arthritis (based on the presence of inflammatory bowel disease [IBD] and arthritis/sacroiliitis) [8–11]. In SpA patients, the positivity of HLA-B27 and the presence of psoriasis, IBD, uveitis, dactylitis, and enthesitis (according to the Leeds enthesitis index [LEI]) were also recorded.

### 2.3. Questionnaire inquiring the pandemic period

A standard questionnaire was applied to the patients via phone call. The phone calls were made in June 2020. Accordingly, the following information questioned for the period between March 10, 2020, and the day of phone call: the presence of any signs of coronavirus infection; whether or not being diagnosed with COVID-19; if diagnosed with COVID-19, the place (hospital, home) where the patient was followed up; whether or not being quarantined due to COVID-19 infection; whether or not having biological or synthetic DMARDs on hand; whether or not the medications were administered during this time; if not administered, the reason(s); whether or not being contacted with his/her physician; and the current disease activity. For the assessment of the current general health status and disease activity, the patients were asked about their general health status (global patient assessment) and to rate it from 0 (excellent) to 10 (very bad), and about current disease activity, the patients were asked to rate their disease activity as “completely under control”, “mild”, “moderate”, “active”, and “highly active”.

### 2.4. Assessments in the study

In the present study, for the patients who discontinued their bDMARDs, comparisons were performed for the following parameters: age (also categorized in decades), sex, mean disease duration (categorized as 1, 5, and 10 years), seropositivity, sequence of use of bDMARDs (bDMARD-naïve, second-line, third-line), and usage of anti-TNF versus nonanti-TNF bDMARDs. The disease activity scores within the 3 months before the declaration of the pandemic was also recorded; these included mean DAS-28 score, CDAI, and SDAI scores (patients were dichotomized as those with and without low disease activity according to the DAS-28, SDAI, and CDAI scores), mean HAQ score (patients were dichotomized according to the scores of 0.5 and 1), mean BASDAI, BASFI, and ASDAS-CRP scores (patients were grouped according to ASDAS-CRP score), CRP level (<5 mg/L and >5 mg/L), patients’ global assessments-VAS score (grouping with 10-unit intervals), general health status (completely under control, mild, moderate, severe, highly severe), and presence of suspected COVID-19. Patients’ global assessment-VAS scores before the pandemic were compared with those during the pandemic. Accordingly, an increase by >2 units in the score was defined as worsened disease activity, and a decrease by <2 units in the score was defined as improved disease activity. Remission was defined as a general health status-VAS score of ≤2, whereas active disease was defined as a VAS score of ≥4. 

Our study is compliant with the Helsinki Declaration and approved by both the local ethical committee (Hacettepe University; Approval number: 2020/08-25 (KA-17058)) and the Turkish Ministry of Health (Approval number: 66175679-514.05.01-E.170548).

### 2.5. Patient and public involvement

There is no patient or public involvement in this study.

### 2.6. Statistical analysis

Data analyses were performed using the Predictive Analytics SoftWare (PASW) 18.0 (SPSS Inc., Chicago, IL, USA) for Windows. The variables were investigated using visual (histogram, probability plots) and analytic methods (Kolmogorov–Smirnov, skewness, and kurtosis) to determine whether they are normally distributed or not. The descriptive analysis data were expressed as mean, standard deviation (SD), the median (minimum-maximum), or percentages for categorical variables. Chi-square test or Fisher’s exact test was used for categorical variables. Student’s t-test was used to compare normally distributed variables, while the Mann–Whitney U test was used to compare nonnormally distributed variables. The variables identified with univariate analyses (p < 0.20) were further entered the logistic regression analysis to determine independent predictors of drug discontinuation separately for RA and SpA patients. A p-value of <0.05 was considered statistically significant.

## 3. Results

### 3.1. Demographic and clinical characteristics of the patients

The demographic and clinical data and drug preferences of 1394 patients who participated in the study are presented in Table 1. In the RA group, RF was positive in 267 (62.7%) patients, anti-CCP was positive in 206 (56.4%) patients, and RF and/or anti-CCP were positive in 368 (74.5%) patients. In the SpA group, there were 664 (73.7%) patients with ankylosing spondylitis, 57 (6.3%) patients with nonradiographic SpA, 101 (11.2%) patients with peripheral SpA, 111 (12.3%) patients with psoriatic arthritis, and 21 (2.3%) patients with enteropathic arthritis. Extraarticular signs of the SpA patients were uveitis in 110 (12.5%) patients, IBD in 40 (4.6%) patients, and psoriasis in 141 (16.1%) patients. The HLA-B27 positivity was determined in 333 (59.1%) of 563 patients. Thirty-seven (4.8%) patients had dactylitis, and 120 (19.3%) patients had enthesitis (at least one entheseal region according to the LEI). Of the RA patients, 233 (47.3%) were receiving antitumor necrosis factor (TNF) agents, and 260 (52.7%) were receiving nonanti-TNF bDMARDs. On the other hand, of the SpA patients, 811 (90%) were receiving anti-TNF agents, and 90 (10%) were receiving antiinterleukin (IL)-17 treatment.

**Table 1:** Demographic and clinical characteristics of the patients.

	Patients with RAn = 493	Patients with SpAn = 901
Female sex, n (%)	400 (81.1)	398 (44.2)
Age, years, median (range)	55 (18–86)	43 (20–79)
Disease duration, months, median (range)	131 (2–509)	111 (2–672)
ESR, mm/h, median (range)	16 (1–120)	12 (1–103)
CRP, mg/L, median (range)	3.96 (0.1–98.9)	3.84 (0.1–91.1)
Global assessment of health–VAS score, median (range)	30 (0–100)	25 (0–100)
Pain–VAS score, median (range)	30 (0–100)	20 (0–100)
Fatigue–VAS score, median (range)	30 (0–100)	20 (0–100)
HAQ score, median (range)	0.38 (0–90)	–
Number of swollen joints, mean ± SD	0.58 ± 2.21	0.1 ± 0.71
Number of tender joints, mean ± SD	1.31 ± 3.41	0.29 ± 1.6
DAS-28-ESR score, median (range)	2.55 (0.56–8.16)	–
CDAI score, mean ± SD	7.97 ± 8.92	–
SDAI score, mean ± SD	17.83 ± 20.08	–
BASDAI score, n (%)	–	1.55 (0–9.5)
BASFI score, n (%)	–	1.2 (0–9.7)
Hypertension, n (%)	143 (29.7)	137 (15.4)
Obesity, n (%)	166 (35.3)	220 (24.4)
Diabetes mellitus, n (%)	48 (9.9)	57 (6.4)
Hyperlipidemia, n (%)	71 (15.1)	98 (11.2)
Coronary artery disease, n (%)	20 (4.3)	17 (1.9)
COPD, n (%)	11 (2.4)	3 (0.3)
Asthma, n (%)	26 (5.6)	28 (3.3)
Malignancy, n (%)	5 (1)	8 (0.9)
Presence of at least1 comorbidity, n (%)	186 (38.2)	462 (51.6)
Presence of ≥2 comorbidities, n (%)	121 (24.8)	229 (25.6)
Presence of ≥3 comorbidities, n (%)	167 (34.3)	160 (17.9)
ASDAS–CRP, median (range)	–	1.84 (0–5.2)
Abatacept, n (%)	32 (6.5)	–
Adalimumab, n (%)	89 (18.1)	270 (30)
Certolizumab, n (%)	38 (7.7)	146 (16.2)
Etanercept, n (%)	75 (15.2)	182 (20.2)
Golimumab, n (%)	18 (3.7)	90 (10)
Infliximab, n (%)	13 (2.6)	123 (13.7)
Rituximab, n (%)	40 (8.1)	–
Secukinumab, n (%)	–	90 (10)
Tofacitinib, n (%)	77 (15.6)	–
Tocilizumab, n (%)	111 (22.5)	–
Hydroxychloroquine, n (%)	164 (33.3)	13 (1.4)
Leflunomide, n (%)	117 (23.7)	17 (1.9)
Methotrexate , n (%)	135 (27.4)	57 (6.3)
Sulfasalazine, n (%)	23 (4.7)	71 (7.9)

RA, rheumatoid arthritis; SpA, spondyloarthritis; ESR, erythrocyte sedimentation rate; CRP, C-reactive protein, VAS, Visual Analog Scale; HAQ, Health Assessment Questionnaire; DAS-28, the Disease Activity Score-28; CDAI, the Crohn’s Disease Activity Index; SDAI, the Simple Disease Activity Index; BASDAI, the Bath Ankylosing Spondylitis Disease Activity Index; BASFI, the Bath Ankylosing Spondylitis Functional Index; COPD, chronic obstructive pulmonary disease; ASDAS, Ankylosing Spondylitis Disease Activity Score; SD, standard deviation.

### 3.2. Detecting COVID-19 in the inflammatory arthritis patients receiving bDMARDs

A total of 1353 patients were questioned about COVID-19 status. Of all the patients, 39 (2.8%) had at least one suspicious sign of COVID-19, and 26 (1.9%) visited a healthcare center for this reason (Table 2). Fever (body temperature ≥ 38°C) was the suspicious sign in 14 (1.0%) patients. The PCR test was positive for COVID-19 only in 2 (0.15%) of all patients. Both of these patients were treated at home.

**Table 2:** Results of the questions about COVID-19 status in the study patients during the pandemic.

	All patients n = 1353	RA n = 487	SpA n = 866
Presence of any suspected sign of COVID-19	39 (2.8)	6 (1.2)	33 (3.7)
Admission to a healthcare center for suspected COVID-19	26 (1.9)	5 (1.0)	22 (2.5)
Having PCR testing for COVID-19	21 (1.6)	5 (1.0)	16 (1.8)
Quarantine recommendation for suspected COVID-19	10 (0.73)	0 (0)	10 (1.15)
PCR positivity for COVID-19	2 (0.15)	0 (0)	2 (0.23)
Family history of COVID-19 positivity	9 (0.66)	3 (0.6)	6 (0.7)

### 3.3. Use of biological DMARDs during the pandemic

A total of 1362 patients responded to the question about the continuation of bDMARDs during the pandemic. Overall, 247 (18.1%) patients discontinued their bDMARDs. Sixty-six (13.8%) of the RA patients and 181 (20.5%) of the SpA patients discontinued their bDMARDs (p = 0.003). The distribution of the patients who discontinued/did not receive their bDMARDs is demonstrated in Table 3. Among RA patients, etanercept (%5.4) was the least frequently discontinued bDMARD, whereas tocilizumab (%20.5) was the most frequently discontinued bDMARD. The clinical characteristics and disease activity parameters did not differ between the RA patients who discontinued and those who did not discontinue their bDMARDs. In the SpA patient group, those who discontinued their bDMARDs were younger than those who did not (median age, 40 years [range, 21–73 years] vs. median age, 44 years [range, 20–79 years]; p = 0.005). Moreover, the SpA patients who continued their bDMARDs had lower disease activity. The multivariate analysis revealed that age of <40 years, a poorer general health status, a poorer VAS score, and the suspicion for the presence of COVID-19 were the factors that determine the discontinuation of bDMARD therapy in SpA patients (Table 4).

**Table 3:** Distribution of the patients who discontinued their biological disease-modifying antirheumatic drugs.

	Patients with RA n/N (%)	Patients with SpA n/N (%)
All bDMARDs	66 (14.0)	181 (20.5)
Abatacept	4/31 (12.9)	NA
Adalimumab	15/86 (17.4)	46/264 (17.4)
Etanercept	4/74 (5.4)	38/180 (21.1)
Golimumab	3/18 (16.7)	15/86 (15.6)
Infliximab	2/12 (16.7)	29/118 (24.5)
Certolizumab	3/37 (8.1)	31/146 (21.2)
Rituximab	7/39 (17.9)	NA
Tofacitinib	6/75 (8.0)	NA
Tocilizumab	22/107 (20.5)	NA
Secukinumab	NA	22/89 (24.7)
Hydroxychloroquine	19/157 (12.1)	5/181 (2.8)
Leflunomide	14/113 (12.4)	4/181 (2.2)
Methotrexate	14/134 (10.4)	13/181 (7.2)
Sulfasalazine	2/22 (9.1)	13/181 (7.2)

RA, rheumatoid arthritis; SpA, spondyloarthritis; bDMARDs, biological disease-modifying antirheumatic drugs; NA, not applicable.

**Table 4:** Characteristics of the patients with spondyloarthritis who discontinued their biological disease-modifying antirheumatic drugs

	Patients with SpA who discontinued bDMARDs	Patients with SpA who continued bDMARDs	Univariatep	Odds Ratio95% CI	Multivariatep	Odds ratio95% CI
Age (<40 vs. ≥40)	84 (46.4)	243 (34.6)	0.004	1.64 (1.18–2.28)	0.002	1.76 (1.24–2.51)
General health status						
Completely under control	51 (28.2)	390 (55.8)				Reference
Mild	52 (28.7)	103 (14.7)	<0.001	3.86 (2.48–6.01)	<0.001	3.20 (1.99–5.15)
Moderate	51 (28.2)	153 (21.9)	<0.001	2.55 (1.66–3.92)	0.003	2.03 (1.26–3.27)
Severe	21 (11.6)	41 (5.9)	<0.001	3.92 (2.15–7.15)	0.003	2.65 (1.38–5.10)
Extremely severe	6 (3.3)	12 (1.7)	0.010	3.82 (1.38–10.63)	0.103	2.46 (0.83–7.28)
VAS-PGA, <20 vs. ≥20	38 (21.1)	281 (40.2)	<0.001	2.51 (1.70–3.71)	0.016	1.74 (1.11–2.75)
Suspected COVID-19	12 (6.6)	20 (2.9)	0.019	2.41 (1.16–5.04)	0.136	1.83 (0.83–4.03)

Data are presented as numbers (percentage, %).SpA, spondyloarthritis; bDMARDs, biological disease-modifying antirheumatic drugs; CI, confidence interval; VAS-PGA, visual analog scale-patient global assessment; COVID-19, coronavirus disease 2019.

The data on the reasons for drug discontinuation were available in 186 (75.3%) of 247 patients who discontinued their bDMARDs. Of these patients, 60 (32.2%) discontinued the therapy based on the recommendation of his/her physician, 84 (45.1%) discontinued on their own demand/fear, 13 (6.9%) discontinued due to suspected COVID-19, 8 (4.3%) discontinued due to the lack of disease activity, and 21 (11.3%) discontinued due to other reasons. No difference was determined between RA and SpA patients in terms of reasons for discontinuation of bDMARDs. During the pandemic, 550 patients (213 RA patients and 337 SpA patients) were able to communicate with their physicians. The patients communicated with their physicians through phone calls (314 [57.1%] patients), face-to-face interview (203 [36.9%] patients), text message (39 [7.1%] patients), e-mail (19 [3.5%] patients), healthcare staff (assistant, nurse) (13 [2.4%] patients), and relatives (5 [0.9%] patients). In 425 (77.3%) of 550 patients, their physicians recommended them to continue bDMARD therapy. In 37 (6.7%) of 550 patients, their physicians recommended them to receive bDMARDs on demand and/or to extent drug application intervals.

### 3.4. Disease activity during the pandemic

Evaluation of the disease activity during the pandemic in all patients revealed that the disease was completely under control in 46.8% of the patients (in 40.8% of the RA patients and in 50.0% of the SpA patients), whereas 19.1% of the patients (21.7% of the RA patients and 17.7% of the SpA patients) had mild disease activity, 24.7% of the patients (27.9% of the RA patients and 23.1% of the SpA patients) had moderate disease activity, 7.0% of the patients (6.8% of the RA patients and 7.1% of the SpA patients) had active disease, and 2.4% of the patients (2.9% of the RA patients and 2.1% of the SpA patients) had very high disease activity (p = 0.016). The mean general health status-VAS score in all patients during the pandemic was 3.1 ± 2.5; it was 3.4 ± 2.6 in the RA patients and 2.9 ± 2.5 in the SpA patients (p < 0.001). The mean general health status-VAS score in all patients in the prepandemic period was 3.2 ± 2.5; it was 3.6 ± 2.6 in the RA patients and 3 ± 2.4 in the SpA patients (p < 0.001).

As compared with the period before the pandemic, the ratios of patients with worsened disease activity, those with improved disease activity, those in whom the disease has become active (while in remission), those with ongoing remission, those with ongoing active disease, and those showing remission (while having active disease) in the first 100 days of the pandemic are demonstrated in Table 5. In the RA patients, these variables did not show difference between the patients who discontinued and those who did not discontinue their bDMARDs. In the SpA patients, the ratio of those who remained in remission before and during the pandemic was 32.1%. The rate of drug discontinuation was lower in the SpA patients who remained in remission (22.0% in those who discontinued bDMARDs and 34.8% in those who did not discontinue bDMARDs; p = 0.002); other parameters showed no difference between the patients who discontinued and those who did not discontinue bDMARDs.

**Table 5:** As assessed according to the prepandemic period, changes in disease activity determined by the general health status-Visual Analog Scale scores during the pandemic.

Status	Definition	Patients with RAn = 428	Patients with SpAn = 809
Worsened disease activity during the pandemic	>2 units increase in the VAS score after the pandemic as compared with before the pandemic	100 (23.4)	137 (16.9)
Patients with improved disease activity during the pandemic	<2 units decrease in the VAS score after the pandemic as compared with before the pandemic	89 (20.8)	118 (14.6)
While in remission before the pandemic, becoming active during the pandemic	A VAS score of ≤2 before pandemic and ≥4 after the pandemic,	69 (16.1)	100 (12.4)
Remission both before and during the pandemic	A VAS score of ≤2 before the pandemic and ≤2 after the pandemic	91 (21.3)	260 (32.1)
Active disease both before and during the pandemic	A VAS score of ≥4 before the pandemic and ≥4 after the pandemic	121 (28.3)	175 (21.6)
Active disease before the pandemic, remission during the pandemic	A VAS score of ≥4 before the pandemic and ≤2 after the pandemic	52 (12.1)	86 (10.6)

Data are presented as numbers (percentage, %).RA, rheumatoid arthritis; SpA, spondyloarthritis; VAS, visual analog scale; bDMARDs, biological disease-modifying antirheumatic drugs; NA, not applicable.

## 4. Discussion

The COVID 19 pandemic, which was announced in March 2020 by the WHO, has directly influenced the daily life of both healthy individuals and individuals with chronic illnesses^1^World Health Organization (2020). The director-general’s opening remarks at the media briefing on COVID-19 [online]. Website https://www.who.int/dg/speeches/detail/who-director-general-s-opening-remarks-at-the-media-briefing-on-covid-19---11-march-2020. [accessed 27 September 2020]).. Immunosuppressed patients rank first among the patient groups influenced by the pandemic most. Biological DMARDs, which have been used in the last two decades, have been the group of medications primarily focused on due to their potential to increase the risk of infection. The American College of Rheumatology (ACR) and the European League Against Rheumatism (EULAR), which are among the international expert societies on rheumatology, have recommended identification of the risk groups and continuation of bDMARDs as long as possible within the frame of patient–physician communication [4,5]. However, the pandemic has caused severe anxiety and fear in some patients. Uncertainty and fear were more prominent particularly in the early period of the pandemic.

The present study investigated the therapeutic approaches in the first 100 days after the declaration of the pandemic in inflammatory arthritis patients known to receive bDMARD therapy. It was observed that the rate of confirmed COVID-19 cases (0.15%) was extremely low in the first 100 days and that nearly 3% of the patients needed to be evaluated for suspected COVID-19. The first 100 days of the pandemic in Turkey was when a strict lockdown was implemented particularly for the people over the age of 65 and under the age of 18. In that period, people with chronic illnesses in particular were on administrative leave, and many patients self-quarantined themselves. It is likely that such a low rate of COVID-19 determined among the patients with inflammatory rheumatic diseases is associated with the abovementioned strict lockdown. After that period, people began to return to their normal life; therefore, how many of the patients evaluated in the present study will develop COVID-19 infection during their follow-up is an investigation that needs to be performed in the future.

Recent studies supported that patients who take biological and conventional DMARDs have less morbidity in the case of COVID-19 [2]. Moreover, in another study from Turkey consisting of 167 patients with inflammatory rheumatic disease, biologic and conventional DMARDs did not seem to cause worse outcomes [12]. However, overall, 18% of all the inflammatory arthritis patients discontinued bDMARDs in the first 100 days of the pandemic. It was observed that drug discontinuation was more common, particularly in the SpA patients, than in the RA patients. Etanercept was the least frequently discontinued bDMARD in the RA patients. It was understood that etanercept has been used for longer than 20 years and thus considered a relatively reliable therapeutic option for both patients and physicians. The main reason for drug discontinuation was the patients’ fear of bDMARD therapy; on the other hand, drug discontinuation was recommended by the physicians in one-third of the patients. In Italy, a survey was conducted between February 2020 and April 2020 in 955 rheumatic patients [13]. In that patient group receiving advanced treatment, modification of biological therapy was performed in nearly 6% of the patients, which is quite low as compared with the finding of the present survey study. Accordingly, different cultural factors can be considered determinative. In the present study, among the patients who were able to communicate with their physicians, about 7% were recommended to receive treatment on demand or could extent drug application intervals. Receiving bDMARD therapy on demand or extending drug application intervals is a method implemented by clinicians for a long time in daily practice with efficacy and safety proven in the controlled studies. During the pandemic, the physicians preferred this method in some of their patients. On the other hand, there is a patient group trying to reach their physicians but could not reach them. It was understood that a change occurred in the physician–patient communication during the pandemic. Specific to rheumatology, patients’ methods of reaching their physicians should be dwelled on, and further studies are needed on this subject.

Any factor that might explain bDMARD discontinuation in the RA patients could not be determined. On the other hand, it was observed that the SpA patients with the disease under control were more likely to continue their drugs. This finding is likely to indicate that the SpA patients with the disease under control were more adherent to their medications, and they avoided recurrent exacerbations. Fluctuations in disease activity were observed in the first 100 days of the pandemic as assessed according to the prepandemic period. The rate of remaining in remission during the pandemic for the patients in remission before the pandemic was 21% in the RA group and 32% in the SpA group. It was understood that the patients with high disease activity in the prepandemic period experienced more difficulty in the early period of the pandemic.

The present survey study was conducted through phone interviews. We could reach three-fourth of the patients; the rates of drug discontinuation and COVID-19 might be higher among those that could not be reached; thus, the results need to be evaluated within the scope of this limitation. The fact that the information regarding steroid use was not inquired could be considered another limitation.

In conclusion, nearly one-fifth of the RA and SpA patients recorded in the TReasure registry and known to receive bDMARD therapy in the prepandemic period discontinued their drugs in the first 100 days of the pandemic. The frequency of COVID-19 was found to be low in the first 100 days of the pandemic, which corresponds to a period when a strict lockdown was implemented in Turkey. Further investigations need to be performed to find out what will happen when people return to their normal active life. Although patients’ fear of treatment appeared to be the main factor, the treatment was discontinued or also interrupted due to physicians’ recommendations. These treatment modifications in the early period of the COVID-19 pandemic may appear as a worsened disease activity in time. Treatment approaches need to be monitored closely in the following period.

## Authors’ contributions

All authors contributed equally to conceiving and designing the analysis and collecting the data. UK and EB performed the analysis and wrote the paper; all authors revised and edited the final version of manuscript.

## Informed consent

Our study is compliant with the Helsinki Declaration and was approved by both the local ethical committee (Hacettepe University; Approval number: 2020/08-25 (KA-17058)) and the Turkish Ministry of Health (Approval number: 66175679-514.05.01-E.170548).
